# Evaluation of the Winemaking Characteristics of High Anthocyanin Teinturier Grape Varieties (Lines)

**DOI:** 10.3390/foods15020340

**Published:** 2026-01-17

**Authors:** Hongyan Zhang, Xiaoqian Zhang, Yu Deng, Yaoyuan Zhai, Yuanpeng Du, Yulin Fang, Kekun Zhang, Keqin Chen

**Affiliations:** 1 Heyang Viti-Viniculture Station, Ningxia Helan Mountain’s East Foothill Wine Experiment and Demonstration Station, College of Enology, Northwest A&F University, Yangling 712100, China; zhyan@nwafu.edu.cn (H.Z.); zxqxq1998@nwafu.edu.cn (X.Z.); dy2024056510@nwafu.edu.cn (Y.D.); 2024zyy@nwafu.edu.cn (Y.Z.); fangyulin@nwsuaf.edu.cn (Y.F.); 2 State Key Laboratory of Crop Biology, Collaborative Innovation Center of Fruit & Vegetable Quality and Efficient Production, College of Horticulture Science and Engineering, Shandong Agricultural University, Taian 271018, China; duyuanpeng001@163.com

**Keywords:** teinturier grape, wine, phenols, flavor, color

## Abstract

Teinturier grapes are an important germplasm resource for addressing the insufficient accumulation of anthocyanins in grapes under adverse climatic conditions. To enrich the variety diversity, eight newly bred teinturier grape varieties were used for comparison with the traditional teinturier grape variety “Yan 73”. The results showed that A1 wine exhibits high levels of citric and tartaric acids, while the B2 wine showed elevated levels of malic and succinic acids. The C1, B2, and G1 wines showed higher total phenol, anthocyanin, flavonoid, flavan-3-ol, and tannin content. In the free volatile components of C1 wine, α-phellandrene, methyl salicylate, α-Terpineol, β-Myrcene, isoamylol and ethyl acetate were the primary aroma compounds. Meanwhile, the glycosidically bound aroma components of B2 wine were predominantly dominated by nonanal, benzaldehyde, α-terpineol, hexanal, α-phellandrene, and D-limonene. Compared with Y73, B2 and A1 wines have better phenols, while B2, C1 and B5 wines have better flavors, which provides support for the promotion of new varieties.

## 1. Introduction

Grapes (*Vitis vinifera* L.), as one of the temperate fruit trees with a relatively long history of human domestication, have garnered significant attention for their rich nutritional value and unique flavor profile. Among various grape varieties, teinturier grapes stand out with their dark-colored flesh and exceptional nutritional content, becoming a crucial ingredient in making specialty wines. These varieties are rich in anthocyanins and other phenolic compounds, boasting higher anthocyanin content than most non-teinturier grape varieties [1,2]. During winemaking, these grapes require minimal maceration time to achieve deep, vibrant colors, enhancing both the wine’s depth and richness.

Anthocyanins, a class of phenolic compounds widely found in plants, exhibit antioxidant, free-radical scavenging, antibacterial, anti-inflammatory, and antiviral properties while contributing to their coloration [1]. The biosynthesis pathway of plant anthocyanins comprises two major components: the phenylpropanoid pathway and the flavonoid pathway. Structural genes directly affect the synthesis of anthocyanins by encoding related enzymes involved in the process, including chalcone synthase (CHS), chalcone isomerase (CHI), flavanone 3-hydroxylase (F3H), dihydroflavonoid reductase (DFR), anthocyanidin synthase (ANS), anthocyanidin 3-*O*-glucosyltransferase (UFGT), etc. Notably, UFGT acts as the final enzyme in anthocyanin biosynthesis, catalyzing the glycosylation of anthocyanidins into anthocyanins. Regulatory genes regulate the expression of structural genes at the transcriptional level, with MYBs, bHLHs (basic helix loop helix), and WD40s transcription factors regulating anthocyanin synthesis by forming the MYB-bHLH-WD40 (MBW) complex. MBW complexes have been identified in various plants such as strawberries [3] and sweet cherries [4]. After endoplasmic reticulum synthesis, anthocyanidins undergo post-translational modifications, including glycosylation, methylation, or acylation, before being transported to vacuoles for storage and accumulation [5,6].

In many grape regions around the world, the synthesis of anthocyanins is influenced by natural conditions. In the Barossa Valley of Australia, under the Mediterranean climate, the summer is hot and dry. The continuous high temperature and intensified drought stress has led to a decrease in the total anthocyanin concentration of grapes, resulting in a pale wine color and reduced stability [7]. In some sub-regions of Napa Valley in the United States, the accumulation of anthocyanins in grapes has stagnated or degraded in the later stage of ripening, which may be related to changes in local climatic conditions, such as high temperatures and the duration of sunlight [8]. The Yantai production area is located in the eastern part of Shandong Province, China. It is significantly influenced by the ocean, with mild weather but high precipitation, resulting in poor grape skin coloring [9]. The northwestern region includes Xinjiang, Gansu, Ningxia, and western Inner Mongolia, which experiences dry conditions, large temperature differences between day and night, higher temperatures during the growing season [10], and susceptibility to the degradation of grape anthocyanins. In addition, the high-temperature stress caused by global warming has become a key factor restricting the accumulation of anthocyanins in grape fruits.

In production, the anthocyanin content of grapes can be increased through the improvement of cultivation measures. The optimal temperature range for the activity of anthocyanin synthesis-related enzymes is between 17 and 26 °C, and high temperatures exceeding 35 °C can inhibit the accumulation of anthocyanins [11]. A high nitrogen supply in soil can regulate genes involved in anthocyanin synthesis [12], thereby reducing the biosynthesis of anthocyanins in grapes. Appropriate water deficiency facilitates the accumulation of anthocyanins in grapes [13]. Under water stress conditions, changes in endogenous hormone levels within grapes promote anthocyanin synthesis [14,15]. Base leaf removal increases light exposure and air circulation, positively influencing the accumulation of anthocyanins in fruits [16]. Exogenous plant hormones exert distinct regulatory effects on anthocyanin biosynthesis. Auxin inhibits the synthesis of anthocyanins [17]. Cytokinins [18], ABA treatment [19], and methyl jasmonic acid (MeJA) treatment [17] promote the synthesis of anthocyanins. However, these cultivation practices often require substantial labor input and technical expertise from growers, leading to increased production costs.

The development of new grape varieties could enhance the color profile of wine through genetic improvement. Variety improvement serves as an effective solution to insufficient anthocyanin accumulation in grapes across different production regions. Teinturier grape varieties, which contain higher levels of phenolic compounds, can help address this issue. The introduction of teinturier grape varieties dates back to the 19th century [2], when powdery mildew, root aphids, and mold were introduced to Europe. Disease-resistant grape varieties capable of producing high-quality wine became breeding priorities, gradually making teinturier grape varieties a focal point for researchers. Recent studies have revealed that multiple 408 bp repeats (Grapevine Color Enhancer, GCE) on the VvMYBA1 promoter may be associated with the characteristic red flesh and leaves of teinturier grape varieties [20]. Through T2T genome analysis, Zhang et al. [21] discovered that the VvMYBA1 promoter in the teinturier grape variety “Yan73” contains two additional 408 bp repeat sequences compared to the other two non-teinturier grape varieties. More coloring mechanisms in teinturier grape varieties are being uncovered. Despite strong market demand for red-fleshed grapes, the variety selection remains limited. This study systematically evaluates the fruit quality and winemaking characteristics of multiple red-fleshed grape germplasms identified earlier by our team, aiming to reveal their unique brewing value and potential applications, thereby supporting the development and promotion of new red-fleshed grape varieties in the industry.

## 2. Materials and Methods

### 2.1. Grape and Winemaking

This trial was conducted in 2024. Nine teinturier grape varieties Y73, A1, A2, B2, B4, B5, C1, G1, and G2 were collected from the experimental base at Shandong Agricultural University’s South Campus in Taishan District, Tai’an City, Shandong Province (117.6° E, 36.17° N). The basic information is shown in Table A1. The vineyards received standard water and fertilizer management and loam soil, were located in the warm temperate semi-humid monsoon climate with distinct four seasons and concurrent rainfall and heat, with all cultivars being grown under identical viticultural conditions. No plant growth regulators were applied during the growing season until fruit maturity.

Grapes were manually sorted, destemmed, and crushed before being transferred to 5 L glass jars for fermentation. A 24 h maceration at 20–25 °C was conducted with 50 mg/L SO_2_ and 30 mg/L pectinase (Lallzyme Ex, Lallemand, France). After juice separation, commercial yeast (Angel Yeast, Henan, China) at 200 mg/L was added for alcoholic fermentation. Daily monitoring of specific gravity and temperature was performed. When the specific gravity dropped below 0.996, 60 mg/L SO_2_ was added to terminate fermentation. The wine was then aged for one month at 4 °C before sampling for quality analysis.

### 2.2. DNA Extraction and Analysis

Grape leaves were ground into powder in liquid nitrogen, the CTAB method was used to extract grape leaf genomic DNA, and the primer pair (F: 5′-ATCAGTGAGGGTAACAAAGTCA-3′, R: 5′-CTGAAGACAACCACGTGCAAC-3′) was designed (Sangon, Shanghai, China). The polymerase chain reactions (PCRs) were carried out, with 1.0 μL of DNA template, 1.0 μL of each forward and reverse primer, 12.5 μL of 2 × Phanta Flash Master Mix (Vazyme, Nanjing, China) and 9.5 μL of ddH_2_O, and the agarose gel method was used for testing.

### 2.3. Appearance Quality of Grapes

The transverse and longitudinal diameters of grapes were measured with a digital vernier caliper. The fruit shape index was calculated. Each treatment tested 30 grapes.

The color index of grape fruits was measured using a colorimeter (Konica Minolta) by determining the L*, a*, and b* values at different positions on the equatorial plane of each grape. Calculate the color index of red grapes (CIRG), H and c*. Each treatment tested 30 grapes.

Fruit shape index = longitudinal diameter of fruit/transverse diameter of fruit

CIRG = (180° − H)/(L* + c*)

H = arctan b/a*

c* = [(a*)^2^ + (b*)^2^]^0.5^

### 2.4. Determination of Berry Physicochemical Parameters

After centrifugation of grape juice and wine (4 °C, 8000 rpm, 10 min), the basic physical and chemical indexes were determined by FTIR wine analyzer (Anton Paar, Shanghai, China).

After centrifugation (4 °C, 8000 rpm, 10 min), the CIELab parameters of wine were tested by W100 wine color analyzer (Hanon, Jinan, China) to obtain L* (brightness), a* (green/red), b* (blue/yellow), c* (saturation) and h* (tone angle).

### 2.5. HPLC Analysis of Monomer Acid Content in Wines

The organic acids in grapes were determined by high-performance liquid chromatography (Agilent, Santa Clara, CA, USA). The wine was diluted twice and filtered through a 0.22 µm organic membrane for later use. The mobile phase consisted of 0.005 mol/L sulfuric acid. The concentrations of various organic acids in the samples were calculated based on the chromatogram, with units expressed as g/L. The detailed quantitation of the calibration curves is listed in Table A2.

### 2.6. HPLC Analysis of Monomeric Anthocyanins Compounds in Wine

The monomeric anthocyanins compounds in wine were analyzed using HPLC. Wines were filtered through a 0.22 µm organic filter membrane before analysis. The mobile phase A for anthocyanins consisted of water, acetonitrile, and formic acid in a 32:4:1 ratio, while mobile phase B contained water, acetonitrile, and formic acid in a 16:20:1 ratio. All results were expressed as the content of Malavidin 3-*O*-glucoside (Table A2 and Table A3).

### 2.7. Photometric Determination of Total Phenol, Flavonoid, Anthocyanin, Flavan-3-ol and Tannin in Wines and Antioxidant Capacity

Referring to the determination method of Ju et al. [22], the total phenol content was measured using the Folin-Schonka method, with results expressed as gallic acid; the total tannins were determined by the methylcellulose precipitation method, with results expressed as catechins; the total anthocyanins were measured using the pH differential method, with results expressed as equivalent values of Malavidin 3-*O*-glucoside; the total flavonoids were analyzed using the aluminum chloride colorimetric method, with results expressed as rutin; and the total flavan-3-ols were determined using the *p*-DMAC method, with results expressed as (+)-catechins.

The antioxidant activity of the wines was assessed using DPPH radical scavenging assays, following the modified methods of Ding et al. [23]. Results were expressed as micromolar Trolox equivalents per liter (mM TE/L).

### 2.8. GC-MS Analyses of Free Volatile and Glycosidically Bound Aroma Compounds in Wines

Following the method by Yue et al. [24], 1.00 g NaCl and 5 mL wine were accurately weighed into a headspace vial, into which 10 µL of internal standard 4-methyl-2-pentanol (4M2P, 405.00 mg/L) was added. After equilibrating the sample for 5 min using a shaker (260 rpm, 40 °C), extraction fibers were inserted into the vial. The extraction was conducted for 30 min, followed by connection to a gas chromatography system (Thermofisher, Wilmington, DE, USA). The sample was analyzed at 250 °C for 8 min to detect free volatile components.

For analyzing glycosidically bound aroma compounds, the solid-phase extraction column was activated with 10 mL methanol and 10 mL water. Subsequently, 2 mL wine was added, followed by 5 mL distilled water and 5 mL dichloromethane. Finally, 20 mL chromatographic-grade methanol was used to elute glycosidically bound aroma compounds, with the collected eluate transferred to 50 mL centrifuge tubes. The entire extraction process maintained a flow rate of 2 mL/min. The methanol eluent was vacuum-dried under reduced pressure at 30 °C. Residual substances were thoroughly dissolved in 10 mL citric phosphoric acid buffer (0.2 M, pH = 5), and enzymatic hydrolysis was performed for 16 h at 40 °C using an AR 2000 glucosidase solution. Subsequent steps followed the same procedures as those for detecting free volatile components. This instrument is used to analyze the 52 main aroma substances in wine. The standard curve is shown in Table A4 and Table A5.

### 2.9. Sensory Evaluation of Wines

The tasting panel consisted of 10 experts from masters at the College of Enology, Northwest A&F University (6 males, 4 females, aged 20–28 years old); all of the experts have undergone sensory tasting training and can score the wines professionally and objectively. The rating system consisted of four parts: appearance, aroma, taste and balance, and the total score was 100. Approximately 30 mL of wine was prepared in an International Standards Organization (ISO) wine tasting glass. Sensory evaluation was conducted in a clean, odor-free, and well-lit sensory laboratory.

### 2.10. Statistical Analysis

Excel was used for statistical analysis of experimental data. One-way ANOVA and Tukey’s HSD were performed using SPSS 27.0.1, and different letters indicate significant differences between treatments (*p* < 0.05). When significant differences were found among different varieties, if homogeneity of variance held, Fisher’s least significant difference (LSD) was used for multiple comparison analysis; if homogeneity of variance did not hold, then the Tamheini T2 method was used. Heat mapping was performed using the Metware Cloud (https://cloud.metware.cn); a PCA map and a radar map were drawn using Origin 2024.

## 3. Results

### 3.1. Identification and Analysis of DNA Related to Teinturier Grapes

**Figure A1:**
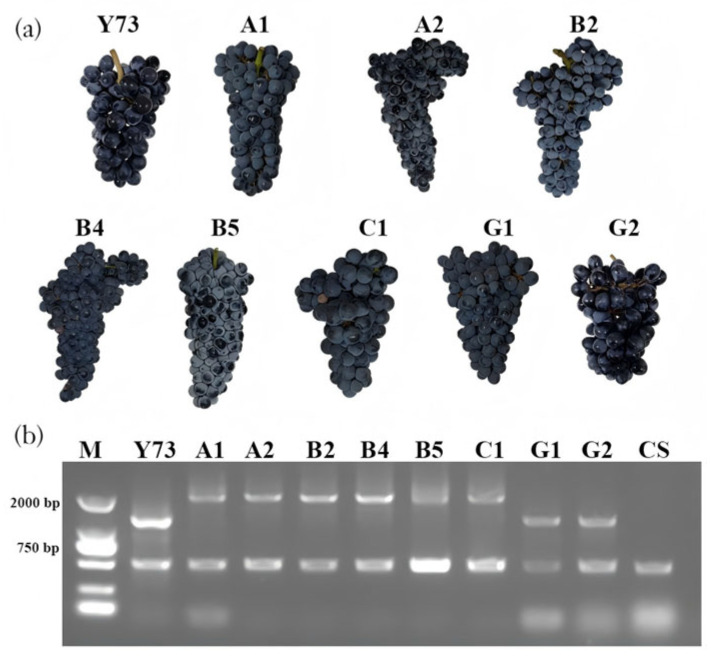
The tandem repeat insertions in the promoter region of VvMYBA1 for different teinturier grape cultivars. (**a**) The grape cluster of nine kinds of teinturier grapes; (**b**) PCR result of the teinturier varieties of DNA prepared from leaves.

Rockel et al. [2] analyzed the VvMYBA1 promoters in teinturier grape varieties and discovered multiple 408 bp DNA sequence repeat insertions in the promoter regions. Moreover, the more inserted fragments there were, the higher the total anthocyanin content in the grape peel and pulp. The research previously assembled the genome of “Yan73” using T2T technology, revealing an 816 bp repeat insertion in the VvMYBA1 promoter of one chromosome of “Yan73” [21]. In this experiment, primers were designed at both ends of the repeat region to analyze grape leaf genomic DNA (Figure A1). Results showed that non-teinturier grape CS (Cabernet Sauvignon) exhibited a single band, while teinturier grape varieties Y73, A1, A2, B2, B4, B5, C1, G1, and G2 displayed multiple bands, indicating the presence of alleles of varying fragment lengths on both chromosomes of teinturier grapes, confirming their hybrid nature. The banding results showed that Y73, G1, and G2 contained three 408 bp repeat fragments, while A1, A2, B2, B4, B5, and C1 had five 408 bp repeat fragments.

### 3.2. Effects of Different Teinturier Grapes on Grape Color

The visual characteristics of grapes include fruit dimensions (transverse and longitudinal diameters), fruit shape index, and coloration. The fruit shape index reflects the roundness of the berries. As shown in Table 1, all eight varieties exhibited significantly smaller longitudinal diameters and fruit shape index compared to Y73. A1 grapes showed smaller transverse and longitudinal diameters, and a relatively low fruit shape index. B2 grapes demonstrated smaller transverse and longitudinal diameters than other teinturier varieties yet maintained a larger fruit shape index. C1 grapes exhibited the smallest fruit shape index. G1 grapes had larger transverse and longitudinal diameters and showed a smaller fruit shape index and a more rounded form.

The L* value indicates brightness in grape coloration, while the a* value represents greenish-red tones and the b* value denotes blue-yellow hues. The hue angle (h*) signifies different color variations, with c* meaning chroma, indicating color vibrancy. The color index of red grape (CIRG) measures redness intensity: lower CIRG values indicate yellowish hues, whereas higher values lean toward deep purple [25]. All eight varieties exhibited surface brightness exceeding Y73, with A1 showing the highest brightness. G1 had a* value < 0, indicating greenish tones, while other varieties predominantly displayed red tones. A1 demonstrated the most intense red hue, compared to G2’s minimal redness. Except for A1, B2, and G2, all other varieties showed yellowish tones. The nine varieties’ h* values ranged from −0.81 to 1.08, with values around zero suggesting dark surface colors. A1 achieved maximum color saturation (c* = 1.18), representing the purest hue, while G2 had the lowest saturation. The CIRG rankings were Y73 > G1 > C1 > B4 > B5 > A2 > G2 > B2 > A1, with CIRG > 6 indicating a bluish-black appearance [26].

### 3.3. Effects of Different Teinturier Grapes on Grape Physiochemical Parameters

The basic physicochemical index measurements of grapes from different cultivars are shown in Table 2. The reducing sugars in grapes primarily consist of glucose and fructose, which serve as the primary substrates for yeast fermentation. Except for B2 and G2 grapes, the reducing sugar content of all grapes is higher than Y73, and the reducing sugar content of A1 and C1 grapes is higher. The contents of total sugar and reducing sugar showed a highly consistent trend. The pH level in grapes is closely associated with acidic compounds. Titratable acid levels are crucial indicators of grape flavor and ripeness. Notably, the titratable acidity of C1 grapes is relatively low, with the highest pH value. The eight cultivar grapes showed significantly lower titratable acidity than Y73, while their pH values were notably higher. In contrast, G2 grapes displayed relatively low levels of reducing sugars, titratable acidity, soluble solids, and pH. These nine cultivar grapes exhibited substantial variations in physicochemical characteristics. The grape pH values ranged between 3.49 and 4.45, showing differences among cultivars but maintaining a relatively narrow variation range.

### 3.4. Effects of Different Teinturier Grapes on Wine Physiochemical Parameters

Physicochemical indicators serve as fundamental criteria for evaluating wine quality. As shown in Table 3, all samples exhibited residual sugar levels between 4.1 and 12.0 g/L, classifying them as semi-dry wines. The pH values ranged from 3.56 to 4.21. Among all tested samples, C1 wine demonstrated higher ethanol content, titratable acidity, pH, and residual sugar levels. Varieties A1 and G1 showed elevated ethanol content with lower values for other parameters, indicating the effective conversion of sugars into alcohol and carbon dioxide during fermentation, demonstrating good sugar utilization efficiency throughout the winemaking process. Varieties B4 and G2 had higher ethanol levels than Y73 but lower residual sugar content compared to others. Significant differences were observed in titratable acidity across varieties: A2 had the highest content while G1 had the lowest. pH values peaked in C1 wine and dipped in A1 wine. In summary, varieties A2 and C1 exhibited higher ethanol content, pH levels, and acidity compared to others.

### 3.5. Effects of Different Teinturier Grapes on Wine Colour

Color is a crucial characteristic of wine, with nine color indicators for teinturier wines shown in Table 4. All samples showed L* values ranging from 2.77 to 29.57, indicating generally low brightness and darker body colors. Significant brightness variations were observed between samples: C1 had the lowest brightness and darkest color, while G2 exhibited the highest brightness and the brightest color. The a* values ranged from 4.19 to 54.48, showing distinct red-tone differences among teinturier varieties. G2 demonstrated significantly higher red tones compared to other samples, with C1 being the lowest. Notably, B2 and C1 wines showed lower red tones than other varieties by more than threefold. The b* values ranged from −1.92 to 18.33, with B5 exhibiting the strongest yellow tones. B2 and C1 wines had negative b* values, indicating bluish tones, while other samples displayed positive values, suggesting yellowish tones.

The c* value indicates the concentration of color intensity. Wine samples showed significant differences with c* values ranging from 4.61 to 55.69, where C1 wine exhibited the lowest color saturation while G2 wine demonstrated the highest saturation and purest hue. h* represents the overall color tendency, with values between −0.43 and 0.40 indicating an overall inclination toward purplish-red or gemstone-red tones characteristic of new red wines [27]. ΔE measures the overall color variation, with all samples ΔE > 3 (ranging from 89.87 to 97.45), suggesting substantial color differences observable to the naked eye [28]. C1 wine showed the most pronounced color difference, while G2 wine had the least. The color parameter analysis revealed that A2, B5, and G2 wines displayed deeper hues with vibrant colors and excellent coloring performance.

### 3.6. Effects of Different Teinturier Grapes on Wine Monomeric Acid Content

**Figure 1:**
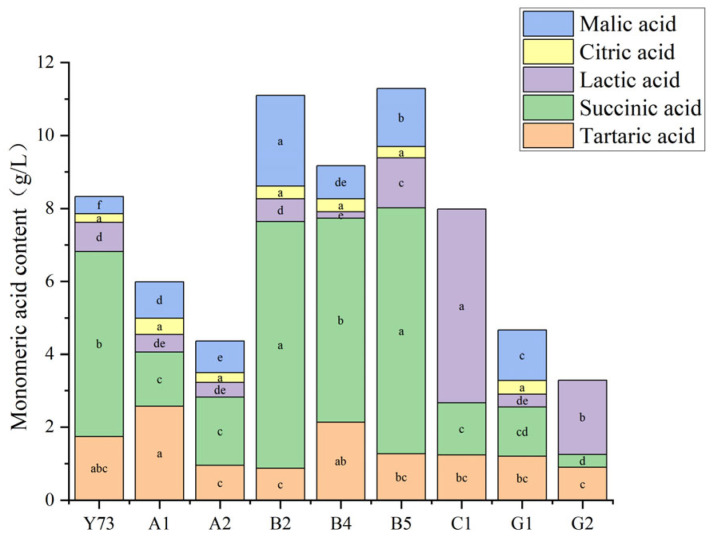
Monomeric acid content of different teinturier grape wines (g/L). Different lowercase letters in the same color grid indicate significant differences (Tukey’s HSD, *p* < 0.05).

The monomeric acids in wine primarily include citric acid, tartaric acid, malic acid, succinic acid, and lactic acid (Figure 1), which play a crucial role in shaping wine flavor [29]. All wine samples contained citric acid levels ranging from 0.24 to 0.45 g/L, meeting the national standard GB/T15037-2006 [30] “Wine” requirement of ≤1.0 g/L. Among the eight test samples, all exceeded the control Y73 wine in citric acid content, with A1 wine showing the highest levels. Tartaric acid, mainly derived from grapes, was present at elevated concentrations in nine samples. A1 wine demonstrated significantly higher tartaric acid content compared to others, while B2 wine had the lowest levels. Malic acid contributed to the wine’s higher acidity, while succinic acid exhibited complex taste profiles and chemical stability. B2 and B5 wines contained notably higher levels of both malic and succinic acids. Lactic acid enhanced wine body richness, and the lactate content of C1 and G2 wines were higher, with that of C1 wine being 2.62–31.24 times higher than other varieties, while B4 wine had the lowest. Overall, teinturier grape varieties contained the highest succinic acid content, followed by tartaric acid, with citric acid being the least abundant. Both C1 and G2 wines showed undetectable malic and citric acids, with succinic and tartaric acid levels remaining relatively low. The significant increase in lactic acid content likely resulted from malolactic fermentation processes in C1 and G2 wines, which consumed malic acid and consequently elevated lactic acid levels.

### 3.7. Effects of Different Teinturier Grapes on Wine Polyphenols Content

#### 3.7.1. Total Phenol, Flavonoid, Anthocyanin, Flavan-3-ol and Tannin in Wines

**Figure 2:**
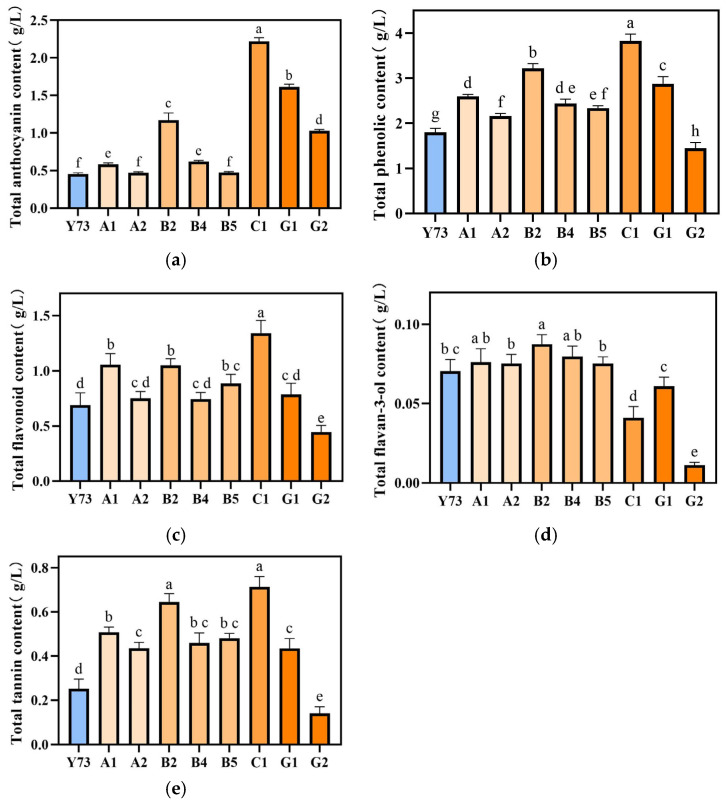
Effect of different teinturier grape on wine total anthocyanins (**a**), phenols (**b**), flavonoids (**c**), flavan-3-ol (**d**) and tannins (**e**). Different lowercase letters indicate significant differences (Tukey’s HSD, *p* < 0.05).

During grape fermentation, phenolic compounds from grapes are extracted into wine through maceration, with their concentration playing a decisive role in determining the wine’s color and flavor profile [31]. This study conducted a comprehensive analysis of polyphenols in wines produced from teinturier grapes, including total phenols, total anthocyanin, total flavonoid, total flavan-3-ol, and total tannin content (Figure 2). The order of the total content of the five phenolic substances in the wines from high to low were C1, G1, B2, A1, B4, B5, A2, G2, and Y73. The content of total anthocyanins, total phenols, total flavonoids and total tannins in C1 wine were higher than other wine samples, corresponding to the lowest L* values in Table 4. B2, A1, and G1 wines contained elevated levels of all phenolic compounds. The A2 wine showed a higher total flavan-3-ol content but lower concentrations of other phenolic components. Notably, the G2 wine demonstrated higher total anthocyanins, while its total phenols, flavonoids, flavan-3-ol, and tannins were significantly lower than those in other samples.

#### 3.7.2. Effects of Different Teinturier Grapes on Wine Monomeric Anthocyanins Content

Anthocyanins are the primary colorants in wine. Table 5 showed the monomeric anthocyanin content in teinturier grape varieties. As illustrated, nine types of monomeric anthocyanins were analyzed, including five basic anthocyanins: Delphinidin 3-*O*-glucoside (Dp), Cyanidin 3-*O*-glucoside (Cy), Petunidin 3-*O*-glucoside (Pt), Peonidin 3-*O*-glucoside (Pn), and Malavidin 3-*O*-glucoside (Mv); two acetylated anthocyanins: Peonidin 3-*O*-(6-*O*-acetyl)-glucoside (Pn-ac) and Malavidin 3-*O*-(6-*O*-acetyl)-glucoside (Mv-ac); and two coumarinated anthocyanins: Trans-Peonidin 3-*O*-(6-*O*-*p*-coumaryl)-glucoside (t-Pn-co) and Trans-Malavidin 3-*O*-(6-*O*-*p*-coumaryl)-glucoside (t-Mv-co).

Among all teinturier wine samples, the predominant anthocyanin was Mv, followed by Mv-ac, with total concentrations of 5038.97 mg/L and 1617.00 mg/L, respectively. This indicated that delphinidin-based anthocyanins were the primary anthocyanins in these nine teinturier grape varieties. The remaining seven anthocyanin monomers ranked from highest to lowest content were Pn, t-Mv-co, Pt, Pn-ac, Dp, t-Pn-co, and Cy. The total monomer anthocyanin content in each wine sample followed this order: C1 > G1 > G2 > B2 > B5 > B4 > A1 > Y73 > A2. Except for Cy, the monomer anthocyanin content in C1 and G1 wines were significantly higher than in other samples. The Cy content in Y73 wine was notably 1.81 times higher than other samples. In comparison with other varieties, the t-Pn-co content in this variety was significantly lower, differing by 0.95 to 3.27 times compared to other wines.

### 3.8. Effects of Different Teinturier Grapes on Wine Antioxidant Activity

**Figure 3:**
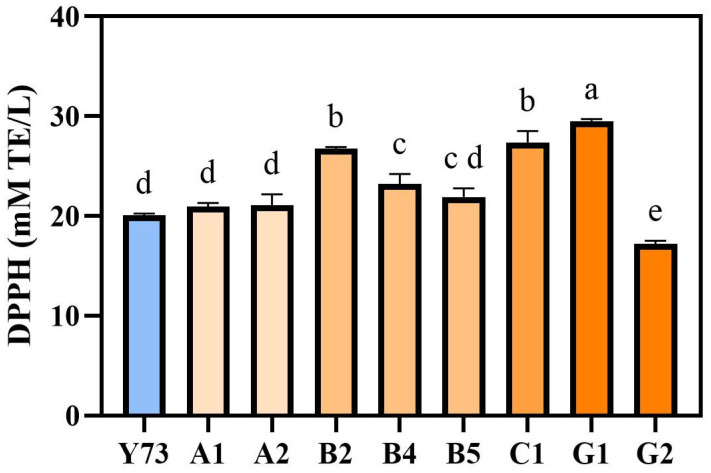
Analysis of antioxidant activity in wines under different teinturier grapes. Different lowercase letters indicate significant differences (Tukey’s HSD, *p* < 0.05).

To evaluate the antioxidant activity in wines of different teinturier grape varieties, the scavenging activity of DPPH free radicals was determined. The scavenging activity range of DPPH free radicals in the nine wines were 16.84–29.67 mM TE/L, respectively (Figure 3). Among them, G1, C1 and B2 wines had relatively high antioxidant activity, while G2 wine had the lowest antioxidant activity. This was similar to the changing trend of phenolic substance content. The phenolic substance content of G1, C1 and B2 wines were relatively high, while the total phenol, total flavonoid, total flavane-3-alcohol and total tannin contents of G2 wine were all lower than those of other wines.

### 3.9. Effects of Different Teinturier Grapes on Wine Aroma

#### 3.9.1. Free Volatile Components in Different Teinturier Grape Wines

**Figure 4:**
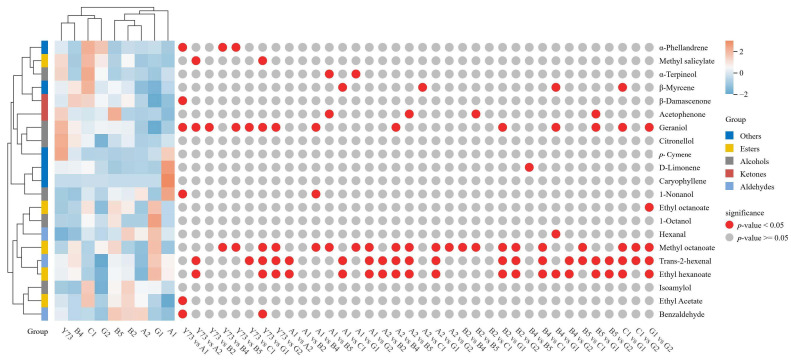
Cluster heat map of free volatile compounds under different teinturier grape wines.

Figure 4 presented the shared free volatile components in wines from different teinturier grape varieties, comprising 21 components, including 6 alcohols, 3 aldehydes, 2 ketones, 5 esters, and 5 other substances. Among all free volatile components in the nine wine samples, the highest concentrations were detected in ethyl acetate, trans-2-hexenal, geraniol, and β-Damascenone. Zhang et al. [32] has demonstrated that alcohols and esters significantly influence the aroma profile of wine. The total aroma contents of the nine wines are C1, B2, B5, G1, Y73, A2, B4, A1 and G2, in sequence.

The heat map reveals the variation trends of free volatile components across different wines. Through cluster analysis, the results indicated that wines can be divided into two distinct categories based on aromatic composition. Y73, B4, C1 and G2 wines belong to the same category. Compared with other wines, the contents of α-phellandrene, methyl salicylate, α-Terpineol, β-Myrcene, isoamylol and ethyl acetate were the highest in the C1 wine. Citronellol, geraniol and *p*-cymene had relatively high contents in the Y73 wine. B5, B2, A2, G1 and A1 wines cluster together. Notably, A1 wine contained elevated 1-nonanol, D-limonene, and caryophyllene, while G1 wine showed higher levels of 1-octanol, methyl octanoate, trans-2-hexenal, ethyl hexanoate, hexanal, and ethyl octanoate. B2, B5, and C1 wine samples demonstrated a higher total aromatic content with a predominance of alcohols and esters, exhibiting rich wines and fruity aromas. In contrast, G2 wine showed the lowest total aromatic content among all varieties.

#### 3.9.2. Glycosidically Bound Aroma Components in Different Teinturier Grape Wines

**Figure 5:**
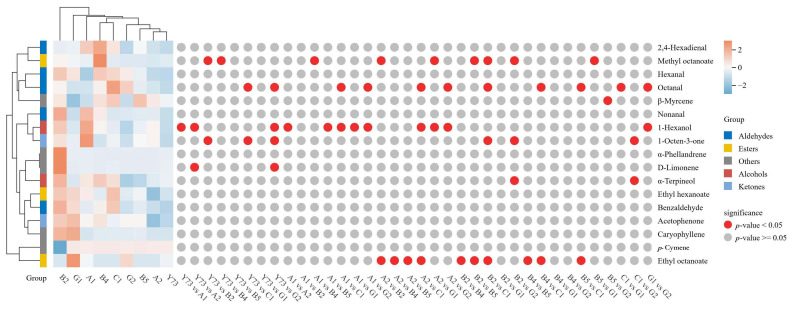
Cluster heat map of glycosidically bound aroma compounds under different teinturier grape wines.

During wine fermentation or aging, glycosidically bound aroma compounds can be hydrolyzed into free-form aromatics through enzymatic or acid hydrolysis, enhancing the wine’s flavor profile. Figure 5 illustrated the shared glycosidically bound aroma compounds across different teinturier grapes, comprising 17 components, including 2 alcohols, 5 aldehydes, 2 ketones, 3 esters, and 5 other substances. Among the nine wine samples, the descending order of aromatic content is hexanal, 1-hexanol, α-terpineol, *p*-cymene, and nonanal.

Cluster analysis revealed three distinct wine categories based on glycosidically bound aroma compounds. The B2 group stood alone as a unique category, exhibiting the highest total aromatic compound content—particularly notable for nonanal, benzaldehyde, α-terpineol, hexanal, α-phellandrene, and D-limonene—surpassing other wine samples. The Y73, G2, A2, and B5 wines formed another cluster with relatively lower total aromatic compounds. Y73 wine showed the lowest concentrations of octanal, nonanal, benzaldehyde, 1-octen-3-one, and caryophyllene. G1, A1, B4, and C1 wines formed the third cluster with moderate aromatic compounds; the contents of ethyl octanoate, acetophenone, *p*-cymene and caryophyllene in the G1 wine sample are all higher than those wines. A1 wine contained elevated levels of 1-hexanol and 1-octen-3-one.

#### 3.9.3. Differences in Wine Aroma Composition

**Figure 6:**
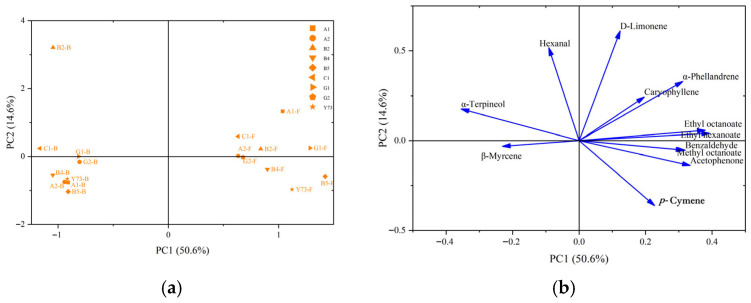
Principal component analysis of aroma components in different teinturier grape wines. (**a**) Score plot; (**b**) loading plot. Abbreviations: F, free volatile compounds; B, glycosidically bound aroma compounds.

Principal component analysis (PCA) was conducted on common free volatile and glycosidically bound aroma components identified in different varieties of wine. As can be seen from Figure 6a, the proportion of PC1 is 50.6%, that of PC2 is 14.6%, and the total proportion of PC1 and PC2 is 65.2%. PC1 contained the information of the vast majority of aroma substances in different types of wines. It can be seen from the distribution positions of the samples that the free volatile components were distributed along the positive axis of the first principal component, while the glycosidically bound aroma compounds were distributed along the negative axis of the PC1.

The principal component analysis of loading plot reflected the overall contribution of aroma substances to different types of wines. As can be seen from Figure 6b, the positive axis of the PC1 was distributed with acetophenone, benzaldehyde, methyl octanoate, ethyl octanoate, ethyl hexanoate, α-phellandrene, D-limonene, *p*-cymene and caryophyllene; its vector direction was highly consistent with the free volatile components data aggregation area of all wines, and these components were the key contributors to the real-time and perceptible free volatile components’ characteristics of wine. The components of α-terpineol, hexanal and β-myrcene were distributed on the negative axis of the PC1, which correspond to the glycosidically bound aroma components position of wines, and these substances constituted the potential aroma of wine. The glycosidically bound aroma components of B2 wine were different from other wines, which was consistent with the clustering results of glycosidically bound aroma components. Among them, the vector positions of hexanal and α-terpineol highly overlaped with those of the B2 wine, indicating that the B2 wine sample was rich in these two glycosidically bound aroma components.

### 3.10. Effects of Different Teinturier Grapes on Wine Sensory Evaluation

**Figure 7:**
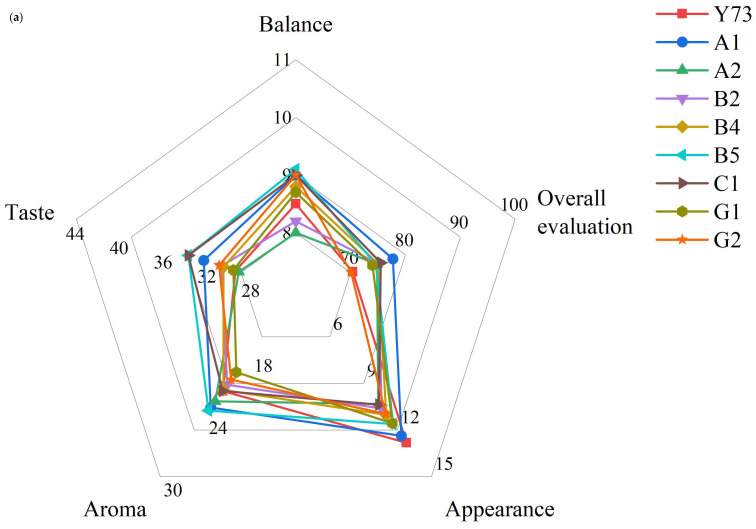

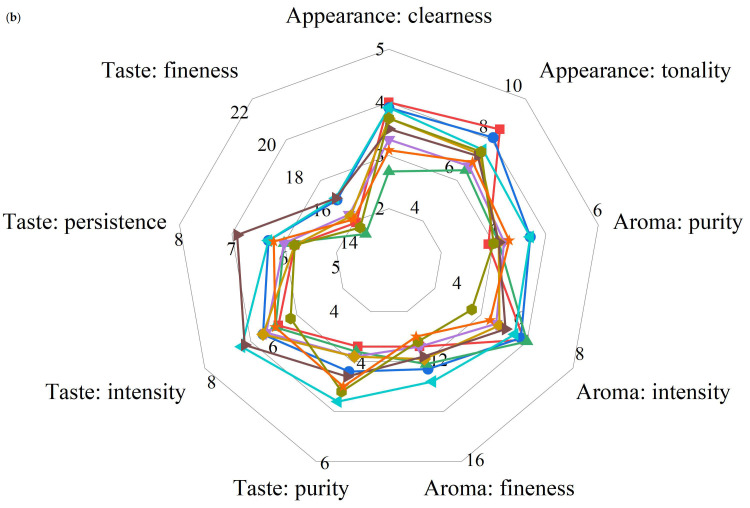
Radar chart for wine sensory evaluation. (**a**) Overall evaluation in different teinturier grape wines; (**b**) sensory evaluation of the appearance, aroma and taste in different teinturier grape wines.

Appearance is the first impression of wine quality, mainly including clearness and tonality. None of the wines scored as high as Y73 in terms of appearance (Figure 7). On the one hand, the L* value of Y73 wine was higher than other wines; on the other hand, it may be related to the low anthocyanin and tannin content, and there was not too much polymer to precipitate. A1 and B5 wines were superior to Y73 in aroma purity and aroma fineness, indicating that their aromas were more pleasant and complex. B5 and C1 wines were superior to Y73 wine in all aspects of taste, which may be because the higher pH value reduced the astringency of the tannins and gave the wine a fuller sense of structure. B5, A1 and C1 performed better in balance, which may be related to their high scores in key indicators (aroma fineness and taste fineness). The sensory quality of wine is the result of the synergistic effect of all its attributes. Compared with the Y73 wine, the overall evaluation of most wines were higher than that of Y73, among which A1 and C1 were the best, while G2 had the same score as Y73, showing no obvious improvement effect.

## 4. Discussion

Anthocyanins, essential components in wine coloration, also exhibit diverse bioactive properties. Studies have shown that anthocyanins have antioxidant capacity [33], anti-inflammatory effects [34], and regulate blood lipids, offering significant benefits for cardiovascular diseases, diabetes, and neurological disorders [35]. Teinturier grape varieties, which contain abundant anthocyanins in both their skins and pulp, demonstrate remarkable advantages in antioxidant properties and health benefits.

Anthocyanins are synthesized through the phenylpropanoid pathway and flavonoid pathway, encoded by structural genes and regulatory genes. In grapes, VvMYBA1 and VvMYBA2 determine the color of the skin. Studies on “Yan73” indicate that anthocyanin accumulation in the pulp tissue may be coordinated by the transcription activator VvMYBA1 and the transcription repressor VvMYBC2-L1 [36]. Lu et al. [37] found that the “Mio Red” variety accumulates substantial anthocyanins in its pulp during ripening, with this accumulation closely associated with the high expression of specific genes such as *DFR* and *ANS*. Analysis of candidate transcription factors also revealed that genes like *ERF23* and *ERFCBF6* participate in the regulatory network of anthocyanin synthesis. In the study of the red-fleshed grape variety “Zhongshan Hongyu”, VvMYBA1 interacts with VvGST4 to activate its promoter activity [38]. The number of GCE repeats in the 408 bp promoter region of the *VvMYBA1* gene in grapes is closely related to anthocyanin content [20]. We discovered that the *VvMYBA1* promoter region on one chromosome of the teinturier grape “Yan73” contains two additional GCE fragments compared to non-teinturier grapes [21]. In this study, PCR technology identified multiple GCE insertions in nine teinturier grape varieties, while the non-teinturier grape variety “Cabernet Sauvignon” contained only one GCE fragment.

Different grape-growing regions have distinct terroir conditions, which may lead to significant differences in the accumulation of anthocyanins in grapes. The Yantai region has a temperate monsoon climate with concurrent rainfall and heat, typically featuring low solar radiation levels. Rainfall during grape ripening accounts for 70% of the annual total rainfall, creating climatic challenges for the accumulation of phenolic compounds in grapes, resulting in low sugar content and insufficient grape skin coloration [39]. The northwest region of China, blessed with abundant sunshine, large diurnal temperature variations, and arid conditions with scarce rainfall, is recognized as the primary area for cultivating wine grapes. However, the high summer temperatures in this region, particularly during grape ripening, reduce the synthesis rate of anthocyanins while accelerating their degradation [40], hindering their accumulation. Therefore, this study has developed several high-anthocyanin content teinturier grape varieties, with total anthocyanin levels in their wines reaching 474.08–2220.24 mg/L, providing solutions to the insufficient anthocyanin accumulation issue in grapes from various regions. In future studies, investigations can be conducted to examine the variations in grape and wine quality under abiotic stress conditions (e.g., drought stress). Such research will facilitate the breeding of novel teinturier grape varieties with enhanced stress tolerance and superior quality attributes, thereby addressing market requirements and driving industrial advancement.

The genetic traits of grapes play a crucial role in their anthocyanin composition, while external factors such as climate, soil types, and cultivation practices also influence the accumulation of anthocyanins in grapes. The interaction between genetic characteristics and terroir conditions is essential for enhancing both grape quality and wine uniqueness. Current research on teinturier grape varieties has been increasing. Multiple studies indicate that light significantly impacts the biosynthesis of anthocyanins. Li et al. [41] found that bagging treatments significantly reduced anthocyanin content in “Kolor” grapes, with more pronounced effects observed in the grape skin. UFGT expression increased in the pulp while decreasing in the skin. Bagging inhibited anthocyanin accumulation in the pulp of “Zhongshan Hongyu,” and the pulp coloration resumed after bag removal [42]. Different wavelengths of light, such as ultraviolet, blue, and red light, also affect anthocyanin biosynthesis, particularly blue light. Wang et al. [5] hypothesized that blue light activates the transcription of *HY5* and *COL2*, subsequently inducing downstream transcription factors like VvMYBA1, VvMYBA2, and structural genes like *CHS, CHI*, and *F3′5′H*, ultimately leading to pulp color transformation in teinturier grapes. Through scientific cultivation practices and technological innovations, grape anthocyanin accumulation can be effectively promoted to enhance fruit quality. However, scientific cultivation control requires specific technical expertise and equipment investment, increasing labor costs and reducing profits. Conversely, variety improvement by selecting suitable grape varieties based on regional characteristics can effectively save labor and capital while boosting vineyard yields and profitability.

There are significant differences in the adaptability and application potential of different teinturier grape varieties, and their unique metabolic characteristics and sensory performance together constitute their own winemaking characteristics. A1 wine had typical characteristics of high phenols, high acidity, high balance, high purity of aroma and high aroma fineness, and may be suitable for making high-quality dry red wine with strong aging potential. A2 wine showed high ethanol content and titratable acidity, and had great coloring characteristics, but its phenolic content was lower than that of other varieties. B2 wine showed outstanding aroma complexity and structural strength. Its malic acid and succinic acid brought a unique sense of acidity layering. The high content of phenolic compounds ensured the excellent fullness and structure of the wine. The content of lactic acid and free volatile components in B4 wine were low. B5 wine was excellent in sensory balance. It had great coloring, high contents of malic acid and succinic acid, and the best evaluation on aroma purity, taste and overall balance. C1 wine had a high alcohol content and acidity, high content of phenols, rich free volatile components and good sensory evaluation. G1 wine had a high content of ethanol and phenolic substances, good color and antioxidant performance, but its acid content, free volatile and glycosidically bound aroma components were not high enough. G2 wine had relatively high lactic acid content, but its phenolic substance content was lower than that of other varieties, and both the content of free volatile and glycosidically bound aroma components and sensory evaluation were at the bottom. Overall, different resources have varying qualities. Specific applications can be made based on their quality performance. In the future, specific technical measures can also be formulated for specific varieties and types of wine to enhance their application value.

The high anthocyanin content of these teinturier grape varieties in this study provides a solution for improving cultivars in grape-growing regions with insufficient anthocyanin accumulation. Due to genetic characteristics, even under identical geographical and climatic conditions, there are significant differences in the quality of wine grapes among different varieties [43,44]. European grape varieties demonstrate higher total phenolic compounds, flavonoids, and flavan-3-ol content than other populations [45,46]. Compared to European grape varieties, Vitis vinifera × Vitis amurensis grapes exhibit higher total anthocyanin characteristics, but their fruits contain lower levels of five basic monomeric anthocyanins [47]. Reports indicate that Cabernet Sauvignon wines are rich in acylated anthocyanins, while Pinot Noir may contain none [48]. Li et al. [49] analyzed eight European red wine grapes and found that anthocyanins, particularly Mv, were most abundant in grape skins and wines. Oliveira et al. [50] discovered that wines made from teinturier grapes like Alicante Bouschet contain the highest levels of Mv. In this study, all nine teinturier grape varieties exhibited high anthocyanin content, with Mv and Mv-ac being the two most abundant monomeric anthocyanins. Different teinturier grape varieties also demonstrate unique characteristics. In this study, the C1 wine exhibited significantly higher levels of total anthocyanins, total phenols, total flavonoids, and total tannins compared to other wine samples, with multiple individual anthocyanins reaching elevated concentrations. The B2 wine samples demonstrated an abundant content of individual phenolic compounds. These findings provide material support for understanding the accumulation patterns of anthocyanins in different grape varieties, offering insights into the comprehensive utilization and mechanistic analysis of teinturier grapes.

## 5. Conclusions

There are differences in the quality of various teinturier grape varieties. A1 and B4 grapes showed higher sugar and acid levels during the harvest periods, while A2 and C1 wines showed elevated ethanol content, pH, and acidity. Among eight teinturier grape wines, their citric acid levels surpassed those in control sample Y73. B2 and B5 wines showed higher malic and succinic acid content, whereas C1 and G2 wines contained elevated lactic acid levels. Eight experimental wines showed higher total phenolic compounds (total anthocyanins, total phenols, total flavonoids, total flavan-3-ol, and total tannins) than the control sample Y73. Except for the A2 wine, all others showed higher total monomeric anthocyanin levels than the control Y73. Analysis of phenolic compounds revealed that C1, B2, and G1 wines contained elevated levels of monomeric anthocyanin compounds and total phenolic substances. Free volatile components of all the wines predominantly consisted of ethyl acetate, trans-2-hexenal and geraniol, while glycosidically bound aroma compounds of all the wines featured higher levels of hexanal, 1-hexanol, α-terpineol, and *p*-cymene, in that order. B2 wines demonstrated the highest combined free volatile and glycosidically bound aroma components, whereas G2 wine had the lowest. In sensory evaluation, B5 wine scored higher in aroma and taste. In conclusion, both A1 and B2 wines have a relatively high content of phenolic substances. Moreover, A1 wine performed better in sensory evaluations, while B2 endowed wine with more abundant monomer acids and aroma components; these grapes have the potential to become materials for enriching the wine varieties made from teinturier grapes.

## Figures and Tables

**Table 1:** Appearance measurements of grapes under different teinturier grapes at harvest.

Cultivar	Transverse Diameter (mm)	Longitudinal Diameter (mm)	Fruit Shape Index	L*	a*	b*	H	c*	CIRG
Y73	13.47 ± 0.78 b	16.24 ± 0.78 a	1.21 ± 0.04 a	26.79 ± 0.66 c	0.53 ± 0.22 b	0.43 ± 0.31 ab	0.65 ± 0.44 ab	0.74 ± 0.25 bc	6.52 ± 0.12 a
A1	11.71 ± 0.86 d	12.23 ± 0.95 e	1.05 ± 0.09 b	28.55 ± 1.04 a	1.00 ± 0.48 a	−0.32 ± 0.57 c	−0.26 ± 0.51 d	1.18 ± 0.52 a	6.07 ± 0.26 c
A2	13.23 ± 0.89 bc	13.62 ± 0.91 cd	1.03 ± 0.03 bc	27.27 ± 0.73 bc	0.57 ± 0.18 b	0.35 ± 0.30 ab	0.48 ± 0.44 b	0.73 ± 0.21 bc	6.41 ± 0.13 ab
B2	11.44 ± 0.66 d	11.81 ± 0.63 e	1.03 ± 0.03 bc	27.67 ± 1.29 b	0.65 ± 0.23 b	−0.11 ± 0.52 c	−0.07 ± 0.64 cd	0.82 ± 0.29 b	6.34 ± 0.30 b
B4	12.77 ± 0.74 c	12.98 ± 0.76 d	1.02 ± 0.02 bc	27.23 ± 0.76 bc	0.52 ± 0.23 b	0.22 ± 0.36 b	0.30 ± 0.64 bc	0.67 ± 0.23 bc	6.44 ± 0.14 ab
B5	13.08 ± 0.52 bc	13.73 ± 0.75 c	1.05 ± 0.04 b	27.19 ± 0.61 bc	0.59 ± 0.24 b	0.34 ± 0.32 ab	0.43 ± 0.51 bc	0.74 ± 0.26 bc	6.43 ± 0.11 ab
C1	13.67 ± 0.89 ab	13.75 ± 0.83 c	1.01 ± 0.03 c	26.85 ± 0.63 c	0.30 ± 0.10 c	0.66 ± 0.29 a	1.08 ± 0.36 a	0.75 ± 0.22 bc	6.49 ± 0.11 ab
G1	14.25 ± 0.83 a	14.53 ± 0.81 b	1.02 ± 0.03 bc	27.26 ± 0.7 bc	−0.14 ± 0.07 d	0.48 ± 0.38 ab	−0.81 ± 0.92 e	0.57 ± 0.27 bc	6.50 ± 0.14 a
G2	13.53 ± 0.85 b	13.85 ± 0.73 c	1.03 ± 0.03 bc	27.87 ± 1.30 ab	0.24 ± 0.23 c	−0.29 ± 0.54 c	−0.48 ± 0.95 de	0.56 ± 0.41 c	6.37 ± 0.32 ab

Data are expressed as the means ± SD (*n* = 30); different lowercase letters in the same column indicate significant differences (Tukey’s HSD, *p* < 0.05).

**Table 2:** Physiochemical parameters of different teinturier grapes.

Cultivar	Reducing Sugar (g/L)	Titration Acid (g/L)	Total Sugar (g/L)	pH Value
Y73	162.87 ± 0.81 f	6.16 ± 0.03 a	188.7 ± 0.79 c	3.49 ± 0.01 g
A1	229.37 ± 1.54 a	3.31 ± 0.01 c	255.60 ± 1.92 a	4.01 ± 0.02 e
A2	211.20 ± 1.04 c	2.68 ± 0.04 d	236.27 ± 0.76 b	4.13 ± 0.01 d
B2	140.97 ± 1.24 h	3.53 ± 0.33 bc	165.27 ± 1.42 e	4.05 ± 0.03 e
B4	207.83 ± 1.51 d	3.76 ± 0.03 b	233.57 ± 1.72 b	4.33 ± 0.03 b
B5	166.47 ± 1.12 e	3.60 ± 0.02 bc	190.97 ± 1.10 c	4.28 ± 0.02 c
C1	225.07 ± 1.31 b	2.87 ± 0.04 d	255.97 ± 1.18 a	4.45 ± 0.02 a
G1	168.13 ± 0.70 e	3.77 ± 0.04 b	189.20 ± 0.95 c	3.61 ± 0.02 f
G2	159.70 ± 0.30 g	3.56 ± 0.01 bc	183.55 ± 0.15 d	3.57 ± 0.00 f

Data are expressed as the means ± SD (*n* = 3); different lowercase letters in the same column indicate significant differences (Tukey’s HSD, *p* < 0.05).

**Table 3:** Physiochemical parameters of different teinturier grape wines.

Cultivar	Ethyl Alcohol/%vol	Titration Acid (g/L)	pH Value	Residul Sugar (g/L)
Y73	2.74 ± 0.05 f	8.63 ± 0.09 e	3.79 ± 0.01 cd	7.10 ± 0.17 a
A1	5.72 ± 0.03 b	5.39 ± 0.03 h	3.56 ± 0.00 f	5.07 ± 0.15 c
A2	4.69 ± 0.08 d	11.77 ± 0.15 a	3.82 ± 0.01 cd	6.90 ± 0.00 a
B2	4.53 ± 0.07 d	10.97 ± 0.13 c	3.88 ± 0.01 b	7.00 ± 0.20 a
B4	2.91 ± 0.06 e	6.34 ± 0.14 g	3.82 ± 0.02 c	7.48 ± 0.40 a
B5	4.53 ± 0.06 d	10.67 ± 0.08 d	3.90 ± 0.02 b	5.83 ± 0.15 b
C1	5.47 ± 0.05 c	11.26 ± 0.08 b	4.21 ± 0.01 a	7.07 ± 0.25 a
G1	6.21 ± 0.08 a	4.85 ± 0.03 i	3.78 ± 0.01 d	4.73 ± 0.21 c
G2	2.75 ± 0.03 ef	7.41 ± 0.02 f	3.74 ± 0.00 e	7.57 ± 0.06 a

Data are expressed as the means ± SD (*n* = 3); different lowercase letters in the same column indicate significant differences (Tukey’s HSD, *p* < 0.05).

**Table 4:** Color indicators of different teinturier grape wines.

Cultivar	L*	a*	b*	c*	h*	ΔE
Y73	13.03 ± 0.06 d	38.77 ± 0.06 c	15.06 ± 0.04 c	41.59 ± 0.07 c	0.37 ± 0.00 b	96.45 ± 0.02 c
A1	8.28 ± 0.39 e	30.58 ± 0.82 e	7.75 ± 0.65 f	31.55 ± 0.96 f	0.25 ± 0.01 c	97.05 ± 0.06 b
A2	14.00 ± 0.07 c	41.21 ± 0.10 b	17.30 ± 0.12 b	44.70 ± 0.13 b	0.40 ± 0.00 a	96.97 ± 0.01 b
B2	3.53 ± 0.03 f	9.71 ± 0.27 f	−0.45 ± 0.06 h	9.72 ± 0.26 g	−0.05 ± 0.01 f	97.00 ± 0.01 b
B4	9.01 ± 0.18 e	32.04 ± 0.37 e	8.77 ± 0.31 e	33.22 ± 0.45 e	0.27 ± 0.01 c	96.91 ± 0.03 b
B5	14.91 ± 0.04 b	42.48 ± 0.09 b	18.33 ± 0.11 a	46.26 ± 0.12 b	0.41 ± 0.00 a	96.89 ± 0.04 b
C1	2.77 ± 0.08 f	4.19 ± 0.12 g	−1.92 ± 0.05 i	4.61 ± 0.10 h	−0.43 ± 0.02 g	97.45 ± 0.04 a
G1	12.52 ± 0.76 d	36.29 ± 1.26 d	4.01 ± 0.42 g	36.51 ± 1.30 d	0.11 ± 0.01 e	94.92 ± 0.20 d
G2	29.57 ± 0.23 a	54.48 ± 0.26 a	11.50 ± 0.01 d	55.69 ± 0.25 a	0.21 ± 0.00 d	89.87 ± 0.03 e

Data are expressed as the means ± SD (*n* = 3); different lowercase letters in the same column indicate significant differences (Tukey’s HSD, *p* < 0.05).

**Table 5:** Monomeric anthocyanins of different teinturier grape wines (measured by Malavidin 3-*O*-glucoside, mg/L).

Cultivar	Basic Anthocyanins					Acetylated Anthocyanins		Coumarinated Anthocyanins	
	Dp	Cy	Pt	Pn	Mv	Pn-ac	Mv-ac	t-Pn-co	t-Mv-co
Y73	7.81 ± 0.54 d	23.12 ± 2.02 a	7.85 ± 0.79 d	41.97 ± 3.60 c	318.78 ± 37.84 bc	43.98 ± 4.81 ab	109.31 ± 12.76 b	8.25 ± 0.76 c	16.35 ± 0.75 c
A1	20.29 ± 1.94 b	6.29 ± 0.20 cd	31.78 ± 4.13 b	58.91 ± 4.49 b	363.86 ± 4.49 b	33.23 ± 1.61 c	104.24 ± 11.15 b	13.52 ± 0.66 ab	32.86 ± 0.93 b
A2	13.87 ± 0.87 c	5.03 ± 0.08 d	20.78 ± 1.77 c	63.75 ± 5.67 b	218.6 ± 20.98 c	34.75 ± 4.61 bc	69.27 ± 3.75 c	17.65 ± 0.74 a	32.00 ± 2.09 b
B2	43.59 ± 0.71 a	8.65 ± 0.16 b	65.95 ± 1.46 a	80.07 ± 1.68 a	722.48 ± 7.57 a	47.98 ± 1.01 a	241.03 ± 3.46 a	16.17 ± 0.26 ab	68.09 ± 0.35 a
B4	9.21 ± 0.19 cd	5.29 ± 0.36 d	19.49 ± 1.70 c	44.11 ± 3.64 c	409.73 ± 25.64 b	30.82 ± 2.58 c	107.26 ± 14.32 b	12.09 ± 0.74 bc	31.94 ± 2.64 b
B5	42.82 ± 0.71 a	8.22 ± 0.31 bc	64.89 ± 1.00 a	79.74 ± 1.52 a	711.32 ± 4.00 a	44.49 ± 1.66 ab	235.93 ± 3.4 a	16.33 ± 0.65 ab	66.61 ± 1.00 a
C1	43.65 ± 1.36 a	8.33 ± 0.86 bc	65.88 ± 2.99 a	90.75 ± 6.33 a	765.80 ± 60.78 a	47.88 ± 2.53 a	256.63 ± 3.84 a	16.70 ± 0.62 a	67.12 ± 3.77 a
G1	44.77 ± 2.27 a	8.48 ± 0.39 bc	69.11 ± 1.97 a	87.77 ± 5.01 a	762.20 ± 52.59 a	46.05 ± 2.41 a	257.46 ± 14.84 a	16.75 ± 2.31 a	69.89 ± 5.56 a
G2	45.31 ± 3.45 a	9.53 ± 0.20 b	66.43 ± 6.07 a	79.90 ± 5.89 a	766.20 ± 55.72 a	52.28 ± 6.69 a	235.88 ± 9.22 a	17.53 ± 3.60 a	68.25 ± 2.25 a

Data are expressed as the means ± SD (*n* = 3); different lowercase letters in the same column indicate significant differences (Tukey’s HSD, *p* < 0.05).

## Data Availability

The original contributions presented in this study are included in the article. Further inquiries can be directed to the corresponding authors.

## Appendix A

**Table A1:** The viticultural information on nine teinturier grape cultivars.

Number	Variety Name	Female Parent	Male Parent	Method of Correction	Spacing in the Rows and Spacing Between Rows
Y73	Yan73	Alicante Bouschet	Muscat Hamburg	Single-dish single-arm	1 m × 2.2 m
A1	MCS1	Cabernet Sauvignon	Mitos	Single-dish single-arm	1 m × 2.2 m
A2	MCS2	Cabernet Sauvignon	Mitos	Single-dish single-arm	1 m × 2.2 m
B2	Tai Zhu	Acolon	Merlot	Single-dish single-arm	1 m × 2.2 m
B4	Tai Mei	Acolon	Merlot	Single-dish single-arm	1 m × 2.2 m
B5	Tai zi	Acolon	Merlot	Single-dish single-arm	1 m × 2.2 m
C1	Zen Zi	Merlot	Mitos	Single-dish single-arm	1 m × 2.2 m
G1	Dai Hong	Cabernet Sauvignon	GM9322	Single-dish single-arm	1 m × 2.2 m
G2	Dai Zhu	Cabernet Sauvignon	GM9322	Single-dish single-arm	1 m × 2.2 m

**Table A2:** Identification and quantification of the monomeric substances in wines by HPLC.

Classification	Analytes	Retention Time	Calibration Curves	R^2^	Linear Range (mg/L)	LOD (mg/L)	LOQ (mg/L)
monomeric acid	Citric acid	9.2	y = 622.72x − 0.2284	0.9997	25–5000	0.41	1.35
	Tartaric acid	9.8	y = 864.7x − 0.0125	0.9997	25–5000	1.34	4.45
	Malic acid	10.9	y = 432.57x − 2.8554	0.9999	25–5000	1.08	3.60
	Succinic acid	13.5	y = 326.14x − 0.0706	0.9996	25–5000	1.08	3.60
	Lactic acid	14.5	y = 427.34x − 12.18	0.9998	25–5000	0.63	2.10
monomeric anthocyanins	Malavidin 3-*O*-glucoside	18.91	y = 0.00002x − 0.1768	0.9998	50–200	0.12	0.41

**Table A3:** Inter-day repeatability of partial analytes in HPLC (*n* = 5 × 3 days).

Classification	Analyte	Nominal Concentration (mg/L)	Experimental Concentration (mg/L)	Accuracy (%)	Inter-Day RSD (%)
monomeric acid	Citric acid	25	26.52684	106.11	2.6
		2000	2027.8462	101.39	1.2
		5000	5031.78212	100.64	5.8
	Tartaric acid	25	27.75632	111.03	5.1
		2000	2003.57492	100.18	1.4
		5000	5011.72649	100.23	1.8
	Malic acid	25	25.97321	103.89	1.9
		2000	2011.75643	100.59	1.6
		5000	5034.83629	100.70	2.6
	Lactic acid	25	23.84759	95.39	2.9
		2000	2112.85740	105.64	1.3
		5000	5028.84641	100.58	2.8
monomeric anthocyanins	Delphinidin 3-*O*-glucoside	50	52.38259	104.77	1.6
		100	113.847332	113.85	4.6
		200	213.14551	106.57	3.3
	Cyanidin 3-*O*-glucoside	50	49.76257	99.53	1.6
		100	99.47242	99.47	2.1
		200	211.27496	105.64	3.6
	Peonidin 3-*O*-(6-*O*-acetyl)-glucoside	50	50.72618	101.45	5.5
		100	105.27963	105.28	4.5
		200	214.85760	107.43	1.3
	Trans-Malavidin 3-O-(6-*O*-p-coumaryl)-glucoside	50	53.46362	106.93	2
		100	111.84736	111.85	4.2
		200	217.36583	108.68	1.4

**Table A4:** Identification and quantification of the aroma substances in wines by GC-MS.

Analytes	Slope	Intercept	R^2^	Linear Range (μg/L)	LOD (μg/L)	LOQ (μg/L)
Ethyl Acetate	28.72183151	62.00349364	0.9982	40–2560	8.75	29.15
Isoamyl butyrate	2.613956182	1.088799909	0.9975	10–640	2.57	8.58
Hexanal	0.436330304	−1.207429816	0.9676	10–640	1.45	4.82
Isoamyl acetate	4.155747184	1.462047996	0.9662	10–640	1.54	5.15
1-Butanol	11.40063854	−5.143623509	0.9887	10–640	2.33	7.75
β-Myrcene	6.572226779	−17.0888405	0.9960	10–640	1.81	6.05
α-Phellandrene	9.442930665	−26.30715237	0.9629	10–640	2.56	8.52
N-Butylcyanoacrylate	10.56113161	−9.232903773	0.9987	10–640	1.18	3.94
Heptanal	1.151034172	1.100261694	0.9735	10–640	0.63	2.11
Isoamylol	17.71006127	21.10712124	0.9984	10–640	2.04	6.79
D-Limonene	7.204079185	−21.86312826	0.9913	10–640	1.19	3.98
Cyclohexane	0.219620016	−0.059722789	0.9994	10–640	2.03	6.76
Ethyl hexanoate	10.16370793	−14.52095964	0.9968	10–640	0.93	3.10
Trans-2-hexenal	1.046898853	0.30991204	0.9834	40–2560	11.45	38.15
1-Pentanol	14.7852144	34.19925809	0.9970	10–640	1.34	4.47
Hexyl acetate	14.56991054	−24.89606421	0.9926	10–640	1.46	4.85
2-Methylpyrazine	0.311363247	−0.74984534	0.9922	10–640	2.53	8.43
*p*-Cymene	58.89797178	−212.7311277	0.9793	10–640	0.70	2.32
Octanal	4.16177065	−5.398980123	0.9967	10–640	1.03	3.44
4-methyl-1-Pentanol	0.419100708	0.577058889	0.9983	10–640	1.02	3.40
Rose oxide	3.903584057	−4.250962621	0.9992	10–640	2.23	7.43
3-Hexen-1-ol	3.822934789	0.456569239	0.9968	10–640	2.39	7.97
Methyl octanoate	30.2867111	−83.05918065	0.9865	10–640	1.06	3.52
Nonanal	0.386707404	−0.213751205	0.9805	10–640	1.85	6.16
L-2-Octanol	8.790893425	−1.523211236	0.9993	10–640	0.63	2.11
2-Hexen-1-ol	0.412279263	0.2052193	0.9989	10–640	2.86	9.52
2,4-Hexadienal	2.05764212	−5.047953032	0.9917	10–640	1.89	6.29
Ethyl octanoate	31.6049875	−76.32695657	0.9958	10–640	1.68	5.59
1-Octen-3-ol	3.92985586	−0.43694163	0.9993	10–640	2.43	8.11
Trans-2-Octenal	5.399814874	−12.28942574	0.9632	10–640	1.57	5.25
2-ethyl-1-Hexanol	7.239032142	0.288917864	0.9984	10–640	1.22	4.06
2,4-Heptadienal	3.061586702	−3.41682021	0.9810	10–640	2.59	8.64
Linalool	3.783849513	1.657317641	0.9971	10–640	1.66	5.53
1-Octanol	3.118756623	−0.756687716	0.9993	10–640	1.21	4.03
Benzaldehyde	1.895352935	−2.105297775	0.9802	10–640	1.26	4.21
Terpinen-4-ol	5.726504728	−6.427456867	0.9989	10–640	1.71	5.70
Caryophyllene	2.013767812	−5.172038446	0.9752	10–640	0.99	3.31
Methyl benzoate	12.64967674	−24.52210362	0.9847	10–640	2.38	7.92
1-Nonanol	3.114660168	−0.8959524	0.9983	10–640	1.48	4.92
Diethyl succinate	0.763965233	−1.098857307	0.9928	10–640	0.61	2.03
Acetophenone	3.091659261	−3.12824877	0.9841	10–640	2.68	8.92
α-Terpineol	1.622010745	−1.599972206	0.9985	10–640	1.96	6.52
2,4-Nonadienal	2.495226599	−5.063519669	0.9748	10–640	0.84	2.79
Citronellol	1.370939903	2.953547874	0.9874	10–640	2.92	9.73
Carvone	2.879921772	−1.487297138	0.9909	10–640	1.63	5.45
Cis-Geraniol	1.245606067	2.80258494	0.9888	10–640	1.83	6.10
Methyl salicylate	4.257560496	−8.562719577	0.9833	10–640	0.47	1.57
Geraniol	1.324735886	3.059142902	0.9904	10–640	2.63	8.75
β-Damascenone	9.313942676	−0.898309756	0.9853	10–640	0.83	2.78
Phenylethyl Alcohol	23.44536421	239.789585	0.9860	10–640	2.19	7.28
Trans-β-Ionone	6.394218207	−4.391426416	0.9999	10–640	1.59	5.29
Perilla alcohol	0.094982029	−0.063624483	0.9978	10–640	2.10	6.99

**Table A5:** Inter-day repeatability of partial analytes in GC-MS (*n* = 5 × 3 days).

Analyte	Nominal Concentration (μg/L)	Experimental Concentration (μg/L)	Accuracy (%)	Inter-Day RSD (%)
1-Butanol	10	11.201	112.01	3.60
	320	315.039	98.45	3.10
	640	625.127	97.68	4.00
3-Hexen-1-ol	10	9.152	91.52	3.20
	320	318.125	99.41	1.70
	640	633.099	98.92	2.00
β-Damascenone	10	8.606	86.06	1.90
	320	345.787	108.06	4.10
	640	656.559	102.59	4.60
1-Octen-3-ol	10	10.467	104.67	3.60
	320	322.456	100.77	6.50
	640	627.467	98.04	3.50
Linalool	10	8.753	87.53	8.86
	320	316.846	99.01	2.60
	640	623.375	97.40	7.09
Phenylethyl Alcohol	10	9.430	94.30	2.66
	320	312.561	97.68	9.97
	640	618.452	96.63	9.85
Hexyl acetate	10	9.746	97.46	1.53
	320	310.376	96.99	0.70
	640	631.725	98.71	7.56
Hexanal	10	11.568	115.68	7.52
	320	305.314	95.41	8.45
	640	658.452	102.88	5.99
Trans-2-hexenal	10	10.957	109.57	3.03
	320	329.857	103.08	6.43
	640	628.471	98.20	6.39
D-Limonene	10	9.672	96.72	8.90
	320	311.784	97.43	1.54
	640	619.32	96.77	2.21
